# Using Genome-Wide Predictions to Assess the Phenotypic Variation of a Barley (*Hordeum* sp.) Gene Bank Collection for Important Agronomic Traits and Passport Information

**DOI:** 10.3389/fpls.2020.604781

**Published:** 2021-01-11

**Authors:** Yong Jiang, Stephan Weise, Andreas Graner, Jochen C. Reif

**Affiliations:** Leibniz Institute of Plant Genetics and Crop Plant Research (IPK), Gatersleben, Germany

**Keywords:** bio-digital resource center, genome-wide prediction, barley, genetic resources, gene bank genomics

## Abstract

Genome-wide predictions are a powerful tool for predicting trait performance. Against this backdrop we aimed to evaluate the potential and limitations of genome-wide predictions to inform the barley collection of the *Federal ex situ Genebank for Agricultural and Horticultural Crops* with phenotypic data on complex traits including flowering time, plant height, thousand grain weight, as well as on growth habit and row type. We used previously published sequence data, providing information on 306,049 high-quality SNPs for 20,454 barley accessions. The prediction abilities of the two unordered categorical traits row type and growth type as well as the quantitative traits flowering time, plant height and thousand grain weight were investigated using different cross validation scenarios. Our results demonstrate that the unordered categorical traits can be predicted with high precision. In this way genome-wide prediction can be routinely deployed to extract information pertinent to the taxonomic status of gene bank accessions. In addition, the three quantitative traits were also predicted with high precision, thereby increasing the amount of information available for genotyped but not phenotyped accessions. Deeply phenotyped core collections, such as the barley 1,000 core set of the IPK Gatersleben, are a promising training population to calibrate genome-wide prediction models. Consequently, genome-wide predictions can substantially contribute to increase the attractiveness of gene bank collections and help evolve gene banks into bio-digital resource centers.

## Introduction

Plant genetic resources are the key to adapting crops to a changing climate, but their actual use to improve crops has remained limited. One major obstacle is that little information is available on their intrinsic value for breeding (Keilwagen et al., 2014). On the one hand, this is due to the lack of phenotypic information. Moreover, for most quantitative traits, the breeding value of a genetic resource cannot be inferred from its *per se* performance. This stimulated pioneering activities in biodiversity informatics to unlock historical phenotypic data from *ex situ* collections to assist the informed selection of gene bank accessions (Keilwagen et al., 2014; Philipp et al., 2018; González et al., 2018a). The consolidation of phenotypic information went hand in hand with recent advances in gene bank genomics (Mascher et al., 2019). This is exemplified by the generation of genetic fingerprints of the entire barley collection (∼22,000) of the *Federal ex situ Genebank for Agricultural and Horticultural Crops* (Milner et al., 2019). Based on the genomic profiles, a core set of 1,000 domesticated barley accessions was identified to represent the diversity space of the entire collection Milner et al., 2019). Moreover, comprehensive historical data on thousand grain weight, flowering time, and plant height, collected over seven decades for about ∼13,000 accessions, has been made available (González et al., 2018a,b). Despite these substantial efforts, the value of the IPK collection for barley breeding needs to be further leveraged with the final goal to convert it from a passive seed repository into an active bio-digital resource center such as the *Arabidopsis* Biological Resource Center (Knee et al., 2011).

To obtain a more comprehensive picture of phenotypic variation for important agronomic traits, Yu et al. (2016) proposed that, as a first step, phenotypic and genomic data of the gene bank material should be combined to calibrate a genome-wide prediction model. Genetic fingerprints can then be used in a second step to predict the phenotypes of entire gene bank collections. Genome-wide prediction has been intensively studied in barley (e.g., Lorenz et al., 2012; Sallam et al., 2015; Schmidt et al., 2016; Thorwarth et al., 2017; Abed et al., 2018; Bhatta et al., 2020; Tsai et al., 2020) focusing on panels of elite breeding lines. Their results highlight the potential, but also the challenges, especially when predicting complex traits in populations that are unrelated to the training population. The genomic diversity of elite populations is much lower than that of gene bank collections of barley (Schmid et al., 2018). It is therefore of interest to study the feasibility of genome-wide predictions in the latter.

Genome-wide prediction has mostly focused on continuous phenotypes and only a few studies have investigated the prediction ability for unordered categorical (e.g., Heuer et al., 2016), binomial (e.g., Technow and Melchinger, 2013), or ordinal categorical traits (e.g., Montesinos-López et al., 2015). For gene bank genomics, predicting unordered categorical traits is of particular interest to complete the taxonomic status reported in the passport records. The assignment of German Warmblood horses into subpopulations by genome-wide prediction has been successfully performed (Heuer et al., 2016). The application of this methodology seems straightforward for gene bank genomics, but has not been tested so far.

Our study makes use of a comprehensive and already published data in barley (González et al., 2018a; Milner et al., 2019) that comprise information on 306,049 high-quality SNPs for 20,454 accessions. The overall goal was to deploy genome-wide prediction to extend the body of phenotypic information available for the IPK collection by focusing on flowering time, plant height, and thousand grain weight as well as on growth habit and row type. In particular, the objectives were to (1) study the potential and limitations of genome-wide prediction of the taxonomic status of barley accessions, (2) investigate the ability to predict complex traits within and among different barley subpopulations, and (3) draw conclusions on the potential use of core collections as training populations in genome-wide prediction.

## Materials and Methods

### Genomic and Phenotypic Data

This study is based on previously published genomic (Milner et al., 2019) and phenotypic information of the barley collection of the *Federal ex situ Genebank for Agricultural and Horticultural Crop Species* of Germany hosted at the *Leibniz Institute of Plant Genetics and Crop Plant Research* (IPK) (González et al., 2018a,b). In total, 22,626 barley accessions of the IPK gene bank were fingerprinted applying genotyping-by-sequencing (GBS; Milner et al., 2019). DNA was digested with PstI and MspI (New England Biolabs), and sequencing was done with Illumina HiSeq 2500. After quality control and filtering (Milner et al., 2019), 20,458 accessions remained, and the missing values were imputed using FILLIN (Swarts et al., 2014) resulting in 306,049 SNPs. In this study we excluded four wild barley accessions, thus in total 20,454 accessions were considered. Among the 20,454 accessions, there are 15,557 spring, 3,691 winter, 147 intermediate/facultative accessions. 1,059 individuals have no information about the growth habit. Regarding row type, the collection comprised 3,823 two-rowed, 7,687 six-rowed, 712 deficiens, 338 intermedium, and 246 labile accessions. A total of 7,648 accessions have no information of the row type (Supplementary Table 1).

The phenotypic information includes data for flowering time (FT), plant height (PH), and thousand grain weight (TGW; González et al., 2018a,b). Phenotyping was done during the regeneration of 12,872 spring and winter barley accessions in the past seven decades. FT stands for the number of days when 50% of the plants reached flowering, which corresponds to the stage Z65 (Zadoks et al., 1974). For winter barley, FT is given in days after the 1st of January of each year. For spring barley, FT was given in days after the sowing date. PH was assessed in cm from the soil surface to the top of the spike, including awns. TGW was determined after seed harvest and expressed in grams on a ∼12.5% grain moisture basis. For spring barley, on average 4.4 data records were available per accession. For winter barley, on average 3.5 data records were available per accession. Linear mixed models were implemented in conjunction with routines for assessment of data quality modeling the phenotype as a function of the genotype, year, and a residual (González et al., 2018a,b). Based on rigorous quality assessment, high heritability estimates were obtained for the three traits exceeding 0.8. Best linear unbiased estimations (BLUEs) of all phenotyped accessions were used for each trait as described in González et al. (2018b).

### Analyses of the Population Structure

Pairwise Rogers’ distances (Rogers, 1972) among all accessions were calculated based on the SNP matrix using the statistical software R (R Core Team, 2019). Principal coordinate analysis (PCoA) (Gower, 1966) was applied based on the Rogers’ distances using the “cmdscale” function in R (R Core Team, 2019), and the first two PCos were plotted against each other in order to portray the potential population structure due to row type status and growth habit of the accessions.

### Genomic Prediction for Growth Habit and Row Type

As the number of intermediate (128) and facultative (19) accessions is very small compared with the number of winter and spring accessions, we decided not to treat them as separate groups in genomic prediction. For simplicity, we merged the intermediate and facultative accessions with the winter accessions without deep biological rationale. Moreover, genomic data was used to cluster the 1,059 individuals that had no growth habit information into spring and winter barley populations. Then, we clustered the 7,648 individuals without row type information into five subpopulations (two-rowed, six-rowed, deficiens, intermedium, and labile). The latter was done for winter and spring barley separately.

We applied the genome-wide prediction method for unordered categorical traits suggested by Heuer et al. (2016). When there are only two groups (e.g., the prediction for growth habit), it is equivalent to genome-wide prediction for binary traits (Montesinos-López et al., 2015). This is easily done with a genome-wide best linear unbiased prediction (GBLUP) model for binary response. When there are *K* groups and *K* > 2 (e.g., the prediction for row type), a “one-versus-all” strategy was recommended in Heuer et al. (2016). That is, for each *i* (1≤*i*≤*K*), the individuals with known group information were regrouped into two classes: those in the *i*-th group and those in the remaining groups. A GBLUP model with binary response is then applied to predict the probability *p*_*i*_ of each individual belonging to the *i*-th group. Finally, the predicted group for each individual is given by *arg*⁡*max*_1≤*i*≤*K*_⁡{*p*_*i*_}. The GBLUP model with binary response was implemented using the R package BGLR (Pérez and de los Campos, 2014).

The accuracy of the growth habit grouping was evaluated by performing 20-fold cross-validation in the population of accessions with known growth habit information. A balanced sampling strategy was applied to divide the entire population into 20 subsets, i.e., the proportion of accessions belonging to each growth habit group in each subset equals the proportion in the entire population. Similarly, the accuracy of row type grouping was assessed by *n*-fold cross-validation where n is the number of individuals in the smallest group with known row type information (*n* = 129 for the winter barley population and *n* = 209 for the spring barley population).

### Genomic Prediction for Flowering Time, Plant Height and Thousand Grain Weight

Genome-wide prediction of FT, PH, and TKW was performed using the GBLUP model (VanRaden, 2008). Briefly, the model is described as follows:

(1) y=X⁢β+g+e,

where *y* is an *n*-dimensional vector of the observed phenotypic values (*n* is the number of genotypes), β is the vector of covariates including the common intercept term as well as (row type) subpopulation effects, *X* is the corresponding design matrix, *g* is the *n*-dimensional vector of the genotypic values and *e* is the residual term. In the model, we assume that β are fixed effects, *g* and *e* are random effects and $g∼N\,\left(0,\sigma_{g}^{2}\,G\right)$ and $e∼N\,\left(0,\sigma_{e}^{2}\,I\right)$, where *G* is the marker-derived additive genomic relationship matrix as in VanRaden (2008), *I* is the identity matrix, $\sigma_{g}^{2}$ and $\sigma_{e}^{2}$ are the corresponding variance components. The mixed model equations for genome-wide prediction were implemented using the R package BGLR (Pérez and de los Campos, 2014), in which the prior of $\sigma_{g}^{2}$ was assumed to be an inverse χ^2^-distribution.

We evaluated the prediction ability of GBLUP using the following scenarios:

1. Five-fold cross-validation within each row type group.
2. Five-fold cross-validation in the combined data set with all row type groups. To investigate the influence of row type subpopulation structure, we implemented two methods: 1) ignoring row types; 2) treating the row type as a fixed covariate. In addition to the overall prediction ability for all accessions, we also calculated the prediction ability for each row type subpopulation. In order to investigate the influence of the geographic origin, we implemented a third method in which the ancestry coefficients estimated by ADMIXTURE (Alexander et al., 2009) were used as fixed covariates. The number of subpopulations was set to 12 which is the same as in Milner et al. (2019).
3. Prediction across row type groups, i.e., using each row type group as the training set to predict the performance of accessions in each of the other groups.
4. Using the accessions in a previously defined core collection comprising 1,000 accessions to predict the remaining individuals.
5. Using all phenotyped accessions to predict the remaining individuals.
6. Using the spring barley accessions to predict the performance of the winter accessions and vice versa.

In scenarios 1 to 5, the genomic prediction was performed for the winter and spring subpopulation separately. In particular, accessions in the core collection were also divided into two subsets according to the growth habit. The prediction ability in scenarios 1 to 4 and 6 was estimated as the Pearson correlation coefficient of the predicted and observed values.

For scenario 5, the prediction accuracy of each individual was assessed by the reliability, which is defined as the squared correlation between the true and predicted genotypic value (Mrode, 2014). More precisely, let *g*_*i*_ be the true genotypic value of the *i*-th genotype (i.e., *g*_*i*_ is the *i*-th entry of the vector *g* in Eq. 1) and ${\hat{g}}_{i}$ be the BLUP of *g*_*i*_, the reliability of ${\hat{g}}_{i}$ is denoted by $r_{i}=c\,o\,r\,\left(g_{i},{\hat{g}}_{i}\right)$. Let

C=[C11C12C21C22]=[X′⁢XX′X   1+G-1⁢σe2⁢/⁢σg2]

be the coefficient matrix of the mixed model equations (MME, Henderson, 1975). Let $\left[\begin{array}{cc} C_{11} & C_{12}\\ C_{21} & C_{22} \end{array}\right]$ be a generalized inverse matrix of *C*. Then, the reliability can be calculated as

$$r_{i}=\sqrt{1-\frac{d_{i}\,{\hat{\sigma }}_{e}^{2}}{{\hat{\sigma }}_{g}^{2}},}$$

where ${\hat{\sigma }}_{g}^{2}$, ${\hat{\sigma }}_{e}^{2}$ are the estimated variance components and *d*_*i*_ is the diagonal element in *C*^22^ corresponding to the *i*-th genotype.

## Results

### Genome-Wide Prediction to Cluster Barley Accessions for Growth Habit and Row Type

**FIGURE 1:**
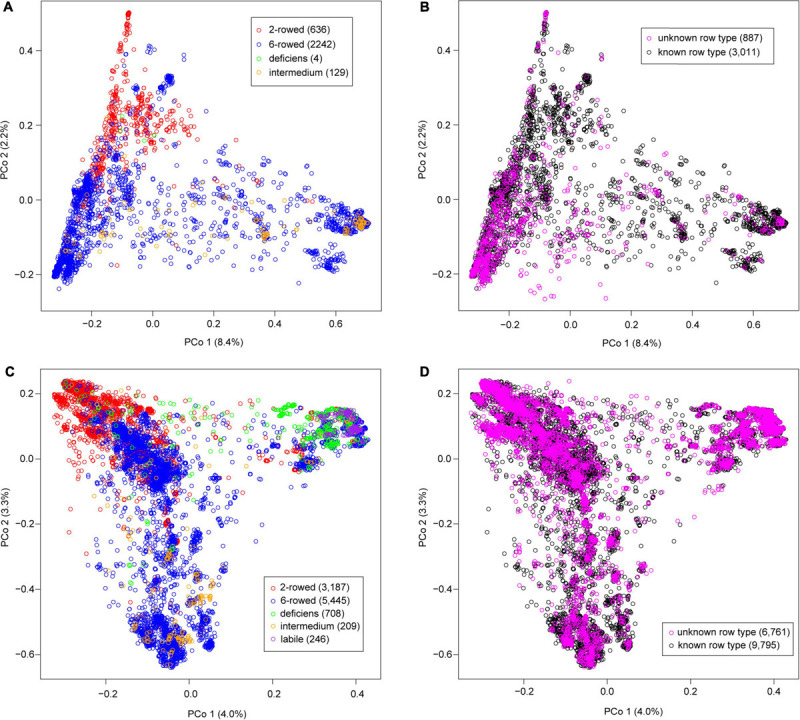
Principal coordinate analysis based on the Rogers’ distance matrix among the 3,898 winter **(A,B)** and 16,556 spring barley accessions **(C,D)**. **(A,C)** The accessions with known row type information were shown in different colors representing the different row types. **(B,D)** The accessions with unknown row type information were added in magenta, while those with known information were recolored as black.

The principal coordinate analysis (PCoA) based on the Rogers’ distance matrix was performed separately for winter (Figures 1A,B) and spring barley accessions (Figures 1C,D). In the winter barley population, the 2-rowed and 6-rowed accessions were clearly separated by the first two PCos despite the small proportion of 10.6% explained variation (Figures 1A,B). The four deficiens accessions were closely related to the 2-rowed accessions, while the intermedium accessions were more closely related to the 6-rowed accessions. In the spring barley population, the first two PCos together explained only 7.3% of the variation (Figures 1C,D). Thus, the stratification by different row types was less clear.

We studied the possibility of using genome-wide predictions to cluster the accessions without prior information on the growth habit and the row type. First, we focused on growth habit and observed in the cross-validation study that on average 78.3% of the genotypes were clustered into the correct growth habit class. Therefore, the whole population with known information on growth habit was used and the 1,059 accessions without prior information were grouped into spring (999 accessions) and winter barley (60 accessions). In the following analyses, spring and winter barley populations were examined separately to classify the accessions into their row type. We first conducted a cross-validation study, which again showed a high prediction ability with on average 91.7% of the genotypes correctly clustered into their row type. Subsequently, the 7,648 individuals without prior information were grouped into row types.

### Phenotypic Accessions Showed a Broad Variation for Flowering Time, Plant Height, and Thousand Grain Weight

**FIGURE 2:**
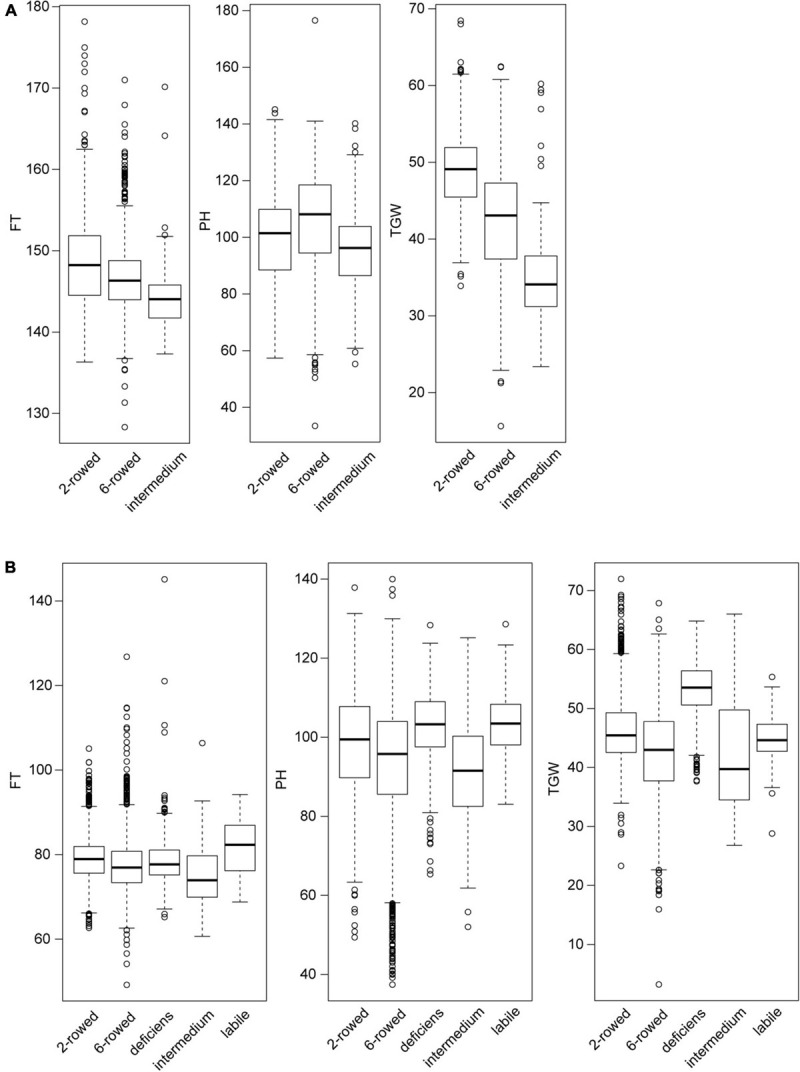
The distribution of phenotypic values for flowering time (FT), plant height (PH), and thousand grain weight for each row type subpopulation of **(A)** winter and **(B)** spring barley accessions. For the box-whisker plots, we used the standard settings of the R function “boxplot.” The horizontal line in the middle of the boxes indicates the median of the data. The boxes extend to the first and third quantile. And the vertical lines extend further to 1.5 inter-quantile range, which is the distance between the first and the third quantiles. All other observed points are plotted as outliers.

Details of the phenotypic data were outlined in a previous study (González et al., 2018b) and we presented therefore only the information, which was relevant for the genome-wide prediction study. Phenotypic data was available for 53% of the spring barley and for 69% of the winter barley accessions that have been genotyped (Supplementary Figure 1 and Supplementary Table 2). For winter barley, we observed substantial differences in the distribution of the phenotypic values in each subpopulation of row types in particular for flowering time and thousand grain weight (Figure 2A). These differences were less pronounced in spring barley (Figure 2B).

### Evaluating the Accuracy of Genome-Wide Prediction of Flowering Time, Plant Height, and Thousand Grain Weight in the Winter Barley Population

We performed genome-wide prediction within and across subpopulation of row types (Scenarios 1 and 3). Since only two accessions belong to the deficiens row type (Supplementary Table 2), they were excluded in the analyses. The within-subpopulation prediction abilities were high with values exceeding 0.6 for all three traits except for flowering time within the intermedium subpopulation (0.45, Table 1). The across-subpopulation prediction abilities were lower than the within-subpopulation prediction abilities. Despite that the size of the 6-rowed subpopulation is nearly 20 times as large as the intermedium subpopulation, using 6-rowed subpopulation as training set to predict the performance of intermedium subpopulation was not better than predicting within the intermedium subpopulation.

**TABLE 1:** Genome-wide prediction abilities within and across row type subpopulations for the winter barley accessions phenotyped for flowering time (FT), plant height (PH), and thousand grain weight (TGW).

**Trait**	**Training set**	**Test set**		
		**2-Rowed**	**6-Rowed**	**Intermedium**
FT	2-Rowed	0.691 (0.013)	0.439 (0.016)	0.260 (0.021)
	6-Rowed	0.544 (0.005)	0.701 (0.007)	0.427 (0.005)
	Intermedium	0.352 (0.103)	0.344 (0.070)	0.450 (0.052)
PH	2-Rowed	0.785 (0.005)	0.593 (0.051)	0.645 (0.020)
	6-Rowed	0.493 (0.014)	0.830 (0.002)	0.712 (0.002)
	Intermedium	0.500 (0.090)	0.532 (0.010)	0.713 (0.021)
TGW	2-Rowed	0.610 (0.012)	0.682 (0.012)	0.582 (0.017)
	6-Rowed	0.354 (0.009)	0.860 (0.002)	0.728 (0.001)
	Intermedium	0.128 (0.101)	0.651 (0.053)	0.741 (0.025)

*The standard deviations of the prediction abilities were presented in brackets.*

We then performed genomic prediction by pooling all accessions (Scenario 2), and observed that the overall prediction abilities for the three traits were high and very similar for the two different methods of treating row type subpopulation (Table 2). The overall prediction ability was mainly driven by the 6-rowed subpopulation as it contains more than 75% accessions of the entire population (Supplementary Table 2). For the other two subpopulations with smaller size (2-rowed and intermedium), the way of treating row types had an influence on the prediction ability: In the 2-rowed subpopulation, the prediction abilities for thousand grain weight in the within-subpopulation scenario and in the pooling scenario modeling row type as covariate were 14% larger than ignoring row types. This may be explained by the significant difference between the mean thousand grain weight values of the 2-rowed and 6-rowed subpopulations (Figure 2A), as well as the clear population stratification revealed by the PCoA (Figures 1A,B). For the intermedium subpopulation, the within-subpopulation prediction accuracy was much lower than the one obtained in the two scenarios pooling all accessions for all three traits. This may be explained by the small size of the subpopulation (∼100 accessions). In particular, the genomic data indicated that the intermedium subpopulation was not clearly separated from the 6-rowed subpopulation (Figures 1A,B). Thus, the prediction ability was increased by treating them as a whole population.

**TABLE 2:** Genome-wide prediction abilities of flowering time (FT), plant height (PH), and thousand grain weight (TGW) obtained by applying five-fold cross validation to the entire set of phenotyped winter barley accessions modeling the row type as covariate (RT).

**Trait**	**Method**	**All**	**2-Rowed**	**6-Rowed**	**Intermedium**
FT	RT-covariate^a^	0.717 (0.004)	0.701 (0.007)	0.703 (0.005)	0.593 (0.032)
	RT-ignored^b^	0.720 (0.004)	0.702 (0.007)	0.703 (0.005)	0.614 (0.030)
	RT-within^c^	n.a.	0.691 (0.013)	0.701 (0.007)	0.450 (0.052)
PH	RT-covariate	0.829 (0.002)	0.797 (0.004)	0.829 (0.003)	0.777 (0.008)
	RT-ignored	0.828 (0.002)	0.798 (0.004)	0.829 (0.003)	0.782 (0.008)
	RT-within	n.a.	0.785 (0.005)	0.830 (0.002)	0.713 (0.021)
TGW	RT-covariate	0.860 (0.002)	0.632 (0.013)	0.859 (0.002)	0.775 (0.018)
	RT-ignored	0.853 (0.002)	0.556 (0.014)	0.854 (0.002)	0.813 (0.012)
	RT-within	n.a.	0.610 (0.012)	0.860 (0.002)	0.741 (0.025)

*^*a*^Row type was treated as a covariate (fixed effect). ^*b*^Row type was not considered in the model. ^*c*^With-in row type prediction, results were extracted from Table 1 for comparison. For each row type subpopulation, the prediction ability was compared with the result obtained in the within-subpopulation prediction. The standard deviations of the prediction abilities were presented in brackets.*

In addition to the row types, the geographic origin of the accessions is also a clear confounding factor in the population structure (Milner et al., 2019). Nevertheless, using the ancestry coefficients estimated by ADMIXTURE instead of row types as fixed covariates did not increase the prediction ability (Supplementary Table 3). In fact, the first two PCs together only explained 5% of the total variation, despite the geographic origins of the accessions can be distinguished (Milner et al., 2019). Thus, our result indicated that the population structure was well exploited by the genomic relationship matrix alone in the GBLUP model.

### Predicting the Genetic Values of Non-Phenotyped Winter Barley Accessions

**FIGURE 3:**
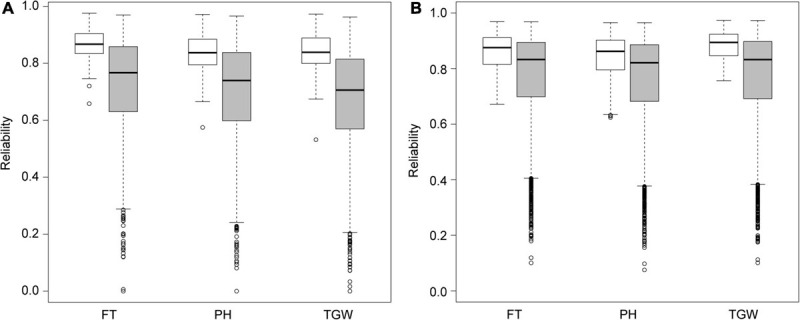
The distribution of reliabilities for the predicted genetic values of **(A)** winter and **(B)** spring barley accessions. Reliabilities for the phenotyped and non-phenotyped accessions were separately presented in white and gray boxes. FT, flowering time; PH, plant height; TGW, thousand grain weight.

Among the 3,898 genotyped winter barley accessions, there were 1,197, 1,216, and 1,791 accessions not phenotyped for flowering time, plant height, and thousand grain weight, respectively. Treating all phenotyped accessions as the training set, we predicted the performances of the non-phenotyped accessions (Scenario 5) using the GBLUP model and estimated the corresponding reliabilities, i.e., the squared correlation between the true and predicted genetic values (Figure 3A). As expected, the reliabilities of the predicted values for the phenotyped accessions were higher than those for the non-phenotyped ones. Nevertheless, the mean reliability of the predicted values for the non-phenotyped accessions were high for all three traits in consideration (flowering time 0.716; plant height 0.690; and thousand grain weight 0.663). There were 78.0, 74.3, and 70.4% of non-phenotyped accessions, whose reliability of predicted values was higher than 0.6 for flowering time, plant height, and thousand grain weight, respectively.

### Evaluating the Accuracy of Genome-Wide Prediction of Flowering Time, Plant Height, and Thousand Grain Weight in the Spring Barley Population

As in the winter barley population, we performed genome-wide prediction within and across subpopulations of row types (Scenarios 1 and 3). Similarly, we observed that the within-subpopulation prediction abilities were high, with values exceeding 0.6 for most trait-subpopulation combinations (Table 3). The across-subpopulation prediction abilities were, in most cases, lower than the within-subpopulation prediction abilities. Exceptions occurred when using the 6-rowed subpopulation to predict flowering time and plant height for the intermedium subpopulation, in which case the prediction ability was increased by 8.7 and 6.3%, respectively. However, the advantage of using the 6-rowed subpopulation as the training set disappeared for the trait thousand grain weight, despite that the size of the 6-rowed subpopulation is about 30 times larger than the intermedium subpopulation.

**TABLE 3:** Genome-wide prediction abilities of flowering time (FT), plant height (PH), and thousand grain weight (TGW) within and across row type subpopulations for the spring barley accessions.

**Trait**	**Training set**	**Test set**				
		**2-Rowed**	**6-Rowed**	**Deficiens**	**Intermedium**	**Labile**
FT	2-Rowed	0.690 (0.003)	0.459 (0.018)	0.527 (0.004)	0.389 (0.053)	0.742 (0.004)
	6-Rowed	0.441 (0.011)	0.741 (0.003)	0.568 (0.002)	0.750 (0.001)	0.834 (0.001)
	Deficiens	0.382 (0.015)	0.342 (0.044)	0.572 (0.032)	0.256 (0.101)	0.817 (0.004)
	Intermedium	0.356 (0.075)	0.460 (0.022)	0.414 (0.048)	0.690 (0.036)	0.451 (0.074)
	Labile	−0.086 (0.176)	0.165 (0.114)	0.447 (0.015)	0.106 (0.131)	0.861 (0.008)
PH	2-Rowed	0.718 (0.003)	0.654 (0.010)	0.348 (0.008)	0.469 (0.028)	0.059 (0.016)
	6-Rowed	0.547 (0.002)	0.815 (0.001)	0.376 (0.005)	0.712 (0.003)	0.429 (0.004)
	Deficiens	0.493 (0.018)	0.417 (0.141)	0.601 (0.013)	0.467 (0.131)	0.255 (0.013)
	Intermedium	0.422 (0.028)	0.493 (0.045)	0.250 (0.018)	0.670 (0.011)	0.141 (0.021)
	Labile	0.006 (0.152)	−0.051 (0.308)	0.341 (0.053)	0.029 (0.277)	0.544 (0.023)
TGW	2-Rowed	0.712 (0.003)	0.706 (0.007)	0.488 (0.006)	0.771 (0.010)	0.004 (0.010)
	6-Rowed	0.325 (0.017)	0.863 (0.002)	0.364 (0.005)	0.754 (0.002)	0.228 (0.005)
	Deficiens	0.358 (0.025)	0.353 (0.159)	0.633 (0.014)	0.205 (0.260)	0.143 (0.005)
	Intermedium	0.287 (0.050)	0.597 (0.042)	0.386 (0.021)	0.852 (0.008)	0.250 (0.016)
	Labile	0.161 (0.180)	0.416 (0.233)	0.219 (0.155)	0.530 (0.384)	0.477 (0.028)

*The standard deviations of the prediction abilities were presented in brackets.*

In the prediction by pooling all accessions together (Scenario 2), we observed that the overall prediction abilities in the entire population were high for the three traits and very similar for the two different methods of treating row types (Table 4), which is similar to the results for the winter barley population. Investigating each row type subpopulation separately, we observed that for the two largest subpopulations (2-rowed and 6-rowed), the prediction ability was similar to the within-population prediction ability and the method of treating row types did not play an important role. For the labile subpopulation, the within-subpopulation prediction ability was higher than the predictability obtained by pooling all accessions. However, the prediction for the other two subpopulations (deficiens and intermediate) benefited from adding the accessions in other subpopulations into the training set and treating the row type as covariates. Compared with the within-subpopulation scenario, the prediction ability for flowering time increased by 11.5% for the deficiens and 12.8% for the intermediate subpopulation, whereas the prediction ability for plant height was increased by 3.7 and 11.8%, respectively. For the thousand grain weight, pooling all accessions also yielded better prediction ability in these two subpopulations than the within-subpopulation prediction scenario. But the way of treating row type had an influence on the prediction ability. In the intermediate subpopulation, we observed the same trend as in the winter barley population that the prediction ability was highest when ignoring the row type, while in the deficiens subpopulation, the highest prediction ability was obtained by treating row type as covariate. This may be explained by the result of PCoA (Figures 1C,D), which showed that the deficiens subpopulation can be distinguished from the two large subpopulations (2-rowed and 6-rowed) whereas the intermediate subpopulation is mixed with the 6-rowed subpopulation. As observed in the winter population, using the ancestry coefficients estimated by ADMIXTURE instead of row types as fixed covariates did not increase the prediction ability (Supplementary Table 4).

**TABLE 4:** Genome-wide prediction abilities of flowering time (FT), plant height (PH), and thousand grain weight (TGW) obtained by applying five-fold cross validation to the entire set of phenotyped spring barley accessions modeling the row type as covariate (RT).

**Trait**	**Method**	**All**	**2-Rowed**	**6-Rowed**	**Deficiens**	**Intermedium**	**Labile**
FT	RT-covariate^a^	0.734 (0.003)	0.685 (0.004)	0.741 (0.003)	0.638 (0.009)	0.779 (0.007)	0.859 (0.004)
	RT-ignored^b^	0.734 (0.003)	0.685 (0.004)	0.741 (0.004)	0.638 (0.008)	0.778 (0.006)	0.859 (0.004)
	RT-within^c^	n.a.	0.690 (0.003)	0.741 (0.003)	0.572 (0.032)	0.690 (0.036)	0.861 (0.008)
PH	RT-covariate	0.794 (0.001)	0.722 (0.002)	0.817 (0.001)	0.623 (0.008)	0.749 (0.007)	0.514 (0.010)
	RT-ignored	0.791 (0.001)	0.719 (0.002)	0.815 (0.001)	0.614 (0.008)	0.751 (0.007)	0.530 (0.011)
	RT-within	n.a.	0.718 (0.003)	0.815 (0.001)	0.601 (0.013)	0.670 (0.011)	0.544 (0.023)
TGW	RT-covariate	0.855 (0.001)	0.719 (0.004)	0.862 (0.001)	0.650 (0.012)	0.849 (0.006)	0.425 (0.019)
	RT-ignored	0.841 (0.001)	0.693 (0.005)	0.850 (0.002)	0.600 (0.011)	0.893 (0.005)	0.349 (0.014)
	RT-within	n.a.	0.712 (0.003)	0.863 (0.002)	0.633 (0.014)	0.852 (0.008)	0.477 (0.028)

*^*a*^Row type was treated as a covariate (fixed effect). ^*b*^Row type was not considered in the model. ^*c*^With-in row type prediction, results were extracted from Table 3 for comparison. For each row type subpopulation, the prediction ability was compared with the result obtained in the within-subpopulation prediction. The standard deviations of the prediction abilities were presented in brackets.*

### Predicting the Genetic Values of Non-Phenotyped Spring Barley Accessions

Among the 16,556 genotyped spring barley accessions, there were 7,758, 7,783, and 9,645 accessions not phenotyped for flowering time, plant height, and thousand grain weight, respectively. As in the winter barley population, we predicted the performances of the non-phenotyped accessions (Scenario 5) using the GBLUP model and estimated the corresponding reliabilities (Figure 3B). The mean reliability of the predicted values for the non-phenotyped accessions was high for all three traits (flowering time 0.783; plant height 0.771; and thousand grain weight 0.781). There were 85.9, 84.3, and 84.9 of non-phenotyped accessions, whose reliability of predicted values was higher than 0.6 for flowering time, plant height, and thousand grain weight, respectively. Compared with the winter barley population, the average reliability for the non-phenotyped spring barley accessions was higher, possibly because of the larger size of training population.

### Predicting Ability From Spring to Winter Barley Accessions and Vice Versa

We observed that the ability of cross-growth-habit prediction (Scenario 6) was generally lower than within-growth-habit prediction for all three traits (Table 5). For plant height and thousand grain weight, the prediction ability from spring to winter barley population was also high (above 0.7), but still 17.8 and 9.1% lower than the prediction ability within the winter barley population. In most cases, the method of treating the row type only played a minor role in the cross-growth-habit prediction.

**TABLE 5:** Genome-wide prediction abilities of flowering time (FT), plant height (PH), and thousand grain weight (TGW) within and across growth habit populations for the entire barley accessions.

**Trait**	**Training set**	**Test set (RT-covariate^a^)**		**Test set (RT-ignored^b^)**	
		**Winter**	**Spring**	**Winter**	**Spring**
FT	Winter	0.717 (0.004)	0.450 (0.031)	0.720 (0.004)	0.465 (0.007)
	Spring	0.154 (0.001)	0.734 (0.003)	0.153 (0.002)	0.734 (0.003)
PH	Winter	0.829 (0.002)	0.373 (0.034)	0.828 (0.002)	0.467 (0.049)
	Spring	0.697 (0.002)	0.794 (0.001)	0.703 (0.001)	0.791 (0.001)
TGW	Winter	0.860 (0.002)	0.473 (0.052)	0.853 (0.002)	0.517 (0.059)
	Spring	0.788 (0.005)	0.855 (0.001)	0.777 (0.006)	0.841 (0.001)

*^*a*^Row type was treated as a covariate (fixed effect). ^*b*^Row type was not considered in the model. The within-population prediction ability was extracted from Tables 4 and 6 for comparison. The standard deviations of the prediction abilities were presented in brackets.*

### Prediction Using the Core Collections

A 1,000 barley core collection was selected from a population consisting of 22,626 accessions with strictly filtered non-imputed marker data (Milner et al., 2019), which is slightly larger than the population of 20,454 accessions with imputed marker data used in this study. A total of 904 out of 1,000 accessions remained in the 20,454 samples. The number of core accessions in each growth habit and row type subpopulation, as well as the number of phenotyped accessions in each group was shown in Supplementary Table 5. We evaluated the accuracy of using phenotyped core accessions to predict the performance of all remaining phenotyped accessions (Scenario 4, Table 6). In the winter barley population, the number of phenotyped core accessions was about 1/10 of the training set size in the scenario of five-fold cross validation, but the prediction ability was only 26.4, 12.0, and 9.9% lower for flowering time, plant height and thousand grain weight, respectively. In the spring barley population, the prediction ability was 28.9, 19.5, and 22.3% lower for the three traits, while the size of training set was only 1/15 of the one in the five-fold cross validation.

**TABLE 6:** The prediction ability of flowering time (FT), plant height (PH), and thousand grain weight (TGW) using the accessions in the core collection as training set and the remaining phenotyped accessions as test set.

**Trait**	**Growth habit**	**Row type**					
		**2-Rowed**	**6-Rowed**	**Deficiens**	**Intermediate**	**Labile**	**All**
FT	Spring	0.512 (0.013)	0.541 (0.007)	0.250 (0.052)	0.608 (0.014)	0.617 (0.047)	0.522 (0.015)
	Winter	0.445 (0.021)	0.540 (0.004)	n.a.	0.182 (0.024)	n.a.	0.531 (0.008)
PH	Spring	0.607 (0.006)	0.684 (0.032)	0.223 (0.039)	0.676 (0.019)	0.113 (0.039)	0.639 (0.023)
	Winter	0.589 (0.014)	0.759 (0.005)	n.a.	0.659 (0.012)	n.a.	0.733 (0.006)
TGW	Spring	0.493 (0.015)	0.651 (0.039)	0.195 (0.115)	0.395 (0.112)	0.083 (0.066)	0.664 (0.037)
	Winter	0.445 (0.033)	0.756 (0.003)	n.a.	0.651 (0.011)	n.a.	0.775 (0.004)

*The standard deviations of the prediction abilities were presented in brackets.*

## Discussion

### Predicting Traits Determining the Taxonomic Status of Gene Bank Accessions

A number of traits are routinely phenotyped during each multiplication cycle of gene bank accessions. This includes 25 phenotypic traits for barley, such as flowering time, plant height, and thousand grain weight, which are subject of this study. In addition, morphological traits are recorded to determine the taxonomic characteristics upon introduction into the gene bank collection and to verify the authenticity of the respective accession during later multiplication cycles. Such descriptor traits include row number, grain hull, awn length or ear color. These are unordered categorical values. The IPK uses the classification system according to Mansfeld (1950) for taxonomic determination.

We used the traits growth habit and row type and investigated the potential of genome-wide predictions to fill the information gaps existing in the IPK barley collection. In line with the findings on subpopulation assignment in German Warmblood horses (Heuer et al., 2016), we observed a high accuracy in predicting the correct class for both traits, which clearly underlines the potential of genome-wide prediction as a promising future tool to support gene bank managers in their tasks of characterizing their collections. Interestingly, the rate of correct classification was much higher for row type (91.7%) than for growth habit (78.3%), which can be explained by a less pronounced subpopulation differentiation for the latter (Figure 1 and Supplementary Figure 1). The larger accuracy in classifying row type versus growth habit may also be explained by less clear phenotype classes for growth habit *versus* row type. A more detailed investigation of the potential of genome-based classification of growth habit can benefit from phenotypic data that will facilitate a deeper description of the growth type. Summarizing, in the case of classification of traits that lead to a strong population differentiation, the description of accessions could be based on predictions alone, otherwise a two-stage strategy with genome-wide predictions followed by validation can be recommended.

An alternative to genome-wide prediction is classification based on functional markers. Key genes are known, which determine the row type such as *Vrs1* and *Vrs5* (for review see Sakuma and Schnurbusch, 2020) or growth habit such as Vrn-H1 (Fu et al., 2005). However, the genomic data of our study are based on genotyping by sequencing. Therefore, prediction based on functional markers is not possible at present, but is deemed feasible with the increasing density of genomic information as it is expected in the future. A further approach to boost the prediction ability for growth habit can be to merge the information of functional markers with genome-wide prediction abilities. For instance, by bridging marker-assisted and genomic selection the prediction accuracy was successfully improved for heading time and plant height in hybrid wheat (Zhao et al., 2014). Similarly, *in silico* determination of the taxonomic status of gene bank accessions will benefit from the ever-increasing number of genes known to be involved in the morphologic and phenologic differentiation of plants.

### Genome-Wide Prediction Is a Powerful Tool for Gene Bank Managers to Increase the Attractiveness of Their Collections

Few studies have explored the potential of genome-wide predictions for gene bank collections with a focus on wheat (Crossa et al., 2016), sorghum (Yu et al., 2016), and cauliflower (Thorwarth et al., 2018). In line with these findings, we also observed very promising results with prediction abilities within subpopulations ranging from 0.45 to 0.86 (Tables 1, 3). In general, the prediction abilities were compatible with the estimated heritabilities in González et al. (2018b). Namely, the prediction abilities were higher for traits with higher heritabilities. This clearly underlines that genome-wide prediction is a powerful tool for gene bank managers.

The optimal strategy for compiling training populations for genome-wide prediction has been intensively discussed in animal and plant breeding (e.g., de Roos et al., 2009; Akdemir et al., 2015). The prediction ability benefits from the size of the training population. Therefore, the combination of data sets from several subpopulations can be attractive to increase the prediction ability. However, this strategy may also reduce the prediction ability if the marker effects vary widely between populations, which is likely to be the case if the subpopulations are not related (de Roos et al., 2009). In line with these findings, we observed that prediction from one row-type subpopulation to another resulted in a strong decrease in prediction abilities compared to a scenario within the subpopulation (Tables 2, 4). This was even more pronounced for the prediction from winter to spring barley and vice versa (Table 5). Interestingly, in most cases, combining data from different subpopulation increased the prediction abilities that were examined within the different subpopulations of row types (Tables 2, 4). Thus, if integrated phenotypic data analysis is feasible, which is not the case for spring and winter barley for the traits under consideration, a joint training population comprising all row type subpopulations may be recommended.

The pros and cons of modeling the subpopulation as cofactor in genome-wide predictions were investigated in our study (Tables 2, 4, 5). We found that considering a cofactor for subpopulations in the case of clear subpopulation differentiation combined with pronounced differences in the population means for the trait under consideration is beneficial. Therefore, these factors should be investigated before deciding which genome-wide prediction model to use.

Previous studies on the potential of genome-wide prediction in dairy cattle (Hayes et al., 2009) and wheat (He et al., 2016) have demonstrated that the prediction ability varies widely between individuals: Individuals, whose genetic background is well represented in the training populations, can be predicted very reliably compared to those who are not well represented. It was therefore recommended to consider not only the predicted value but also the reliability criterion in genomic selection in wheat (He et al., 2016). We observed a large variation in the reliability criterion (Figure 3), which indicates substantial differences in the quality of the predictions in our collection of barley genetic resources. Therefore, both parameters, the predicted value and the reliability criterion should be presented.

### Using a Barley Core Collection as Training Populations

Driven by the still considerable costs, core collections for gene bank collections are often defined for deep sequencing and phenotyping. This was also the case for the IPK barley collection (Milner et al., 2019): A core set of 1,000 accessions was selected, representing the entire molecular diversity of the barley collection. The strategy used is similar to approaches to optimize the calibration set of reference individuals in genome-wide prediction (Rincent et al., 2012). Therefore, it is not surprising that we observed only a moderate decrease in prediction abilities when using the core set compared to the total population (Table 6), considering that we reduced the training population to a fraction of 1/15 of the original number of individuals (Supplementary Table 5). The decrease in diversity can be further reduced if core sets are selected in the context of gene banks using even more efficient criteria for assembling optimized training populations (Rincent et al., 2012). These more advanced strategies should be taken into account in the future assembly of core sets considering also the required sizes of training populations needed to guarantee defined thresholds of prediction accuracies.

## Data Availability Statement

Publicly available datasets were analyzed in this study. The datasets used for this study were published and are available at https://doi.org/10.1038/sdata.2018.278 and https://doi.org/10.1038/s41588-018-0266-x.

## Ethics Statement

All authors declare that this study adheres to ethical standards including ethics committee approval and consent procedure. All experiments were performed under the current laws of Germany.

## Author Contributions

YJ and JR designed the study. YJ performed the data analysis. YJ, SW, AG, and JR wrote the manuscript. All authors agreed with the current statement.

## Conflict of Interest

The handling editor RP and the author AG declared that they are affiliated with the INCREASE consortium on genetic resources in legumes. The remaining authors declare that the research was conducted in the absence of any commercial or financial relationships that could be construed as a potential conflict of interest.
