# Supplementary material for: Using Genome-Wide Predictions to Assess the Phenotypic Variation of a Barley (Hordeum sp.) Gene Bank Collection for Important Agronomic Traits and Passport Information
Source: Front Plant Sci. 2021 Jan 11;11:604781. doi: 10.3389/fpls.2020.604781 (PMC7829250; doi:10.3389/fpls.2020.604781)
Supplement: Supplementary file 1 [file Data_Sheet_1.docx]

Supplementary Material

**Supplementary Table 1:** The number of barley accessions for each row type in the winter and spring barley collection of the IPK Gatersleben before and after genome-wide prediction.

| Row type | Spring original^a^ | Spring predicted^b^ | Winter original^a^ | Winter predicted^b^ |
| --- | --- | --- | --- | --- |
| 2-rowed | 3,187 | 5,948 | 636 | 769 |
| 6-rowed | 5,445 | 8,843 | 2,242 | 2,996 |
| Intermedium | 209 | 237 | 129 | 129 |
| Deficiens | 708 | 1,250 | 4 | 4 |
| Labile | 246 | 278 | 0 | 0 |
| Not classified | 6,761 | 0 | 887 | 0 |
| Total number | 16,556 | 16,556 | 3,898 | 3,898 |

^a^ The row type information according to the original passport data

^b^ The row types of the accessions without information in the passport data were predicted using genomic prediction

**Supplementary Table 2:** The number of spring and winter barley accessions phenotyped for flowering time (FT), plant height (PH), and thousand grain weight (TGW) in each subpopulation of row types.

| Row type | Spring | | | Winter | | |
| --- | --- | --- | --- | --- | --- | --- |
|  | FT | PH | TGW | FT | PH | TGW |
| 2-rowed | 2,761 | 2,756 | 2,157 | 540 | 530 | 389 |
| 6-rowed | 4,894 | 4,877 | 3,823 | 2040 | 2,031 | 1,622 |
| intermedium | 192 | 192 | 119 | 119 | 119 | 94 |
| deficiens | 717 | 714 | 592 | 2 | 2 | 2 |
| labile | 234 | 234 | 220 | 0 | 0 | 0 |
| Total number | 8,798 | 8,773 | 6,911 | 2,701 | 2,682 | 2,107 |

**Supplementary Table 3:** Genome-wide prediction abilities of flowering time (FT), plant height (PH), and thousand grain weight (TGW) using the estimated ancestry coefficients as fixed covariates for the winter barley accessions. The standard deviations of the prediction abilities were presented in brackets.

| Trait | All | 2-rowed | 6-rowed | intermediate |
| --- | --- | --- | --- | --- |
| FT | 0.722 (0.005) | 0.711 (0.007) | 0.702 (0.005) | 0.603 (0.028) |
| PH | 0.830 (0.002) | 0.804 (0.004) | 0.830 (0.003) | 0.785 (0.006) |
| TGW | 0.854 (0.002) | 0.562 (0.013) | 0.855 (0.002) | 0.820 (0.012) |

**Supplementary Table 4:** Genome-wide prediction abilities of flowering time (FT), plant height (PH), and thousand grain weight (TGW) using the estimated ancestry coefficients as fixed covariates for the spring barley accessions. The standard deviations of the prediction abilities were presented in brackets.

| Trait | All | 2-rowed | 6-rowed | deficiens | intermedium | labile |
| --- | --- | --- | --- | --- | --- | --- |
| FT | 0.739 (0.002) | 0.693 (0.004) | 0.746 (0.003) | 0.644 (0.008) | 0.787 (0.006) | 0.853 (0.004) |
| PH | 0.792 (0.001) | 0.720 (0.002) | 0.815 (0.002) | 0.619 (0.008) | 0.751 (0.008) | 0.533 (0.010) |
| TGW | 0.843 (0.001) | 0.695 (0.005) | 0.851 (0.002) | 0.626 (0.010) | 0.900 (0.006) | 0.370 (0.014) |

**Supplementary Table 5:** The number of core accessions for each row type in the winter and spring barley collection of the IPK Gatersleben and the number of phenotyped accessions. FT: flowering time; PH: plant height; TGW: thousand grain weight.

| Row type | Spring | S_FT | S_PH | S_TGW | Winter | W_FT | W_PH | W_TGW |
| --- | --- | --- | --- | --- | --- | --- | --- | --- |
| 2-rowed | 201 | 129 | 129 | 100 | 40 | 29 | 29 | 25 |
| 6-rowed | 429 | 299 | 297 | 222 | 204 | 151 | 148 | 133 |
| Intermedium | 21 | 16 | 16 | 8 | 5 | 4 | 4 | 4 |
| Deficiens | 3 | 3 | 3 | 2 | 0 | 0 | 0 | 0 |
| Labile | 1 | 1 | 1 | 1 | 0 | 0 | 0 | 0 |
| Total | 650 | 448 | 446 | 333 | 249 | 184 | 181 | 162 |


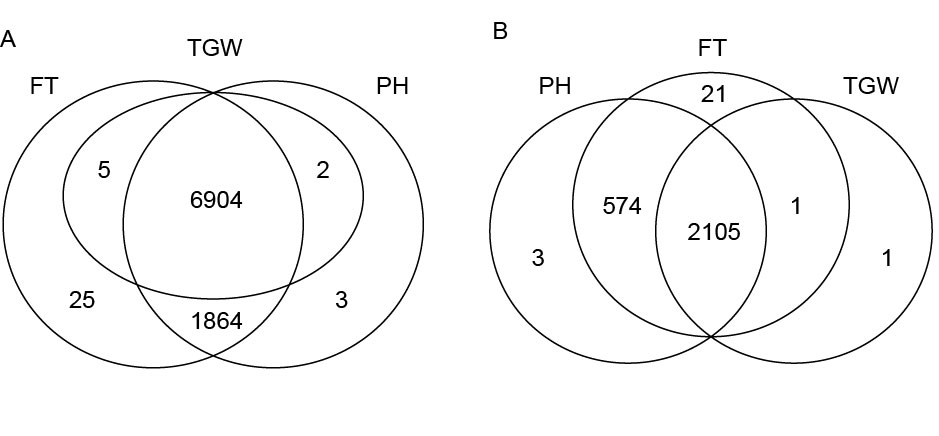


**Supplementary Figure 1:** Venn diagrams showing the number of A) spring and B) winter barley accessions phenotyped for flowering time (FT), plant height (PH) and thousand grain weight (TGW).
